# Antiproliferative Effect of Methanolic Extract of *Vernonia greggii* (Asteraceae) on Human Tumoral HeLa Cells Nanoencapsulated into PLGA-Nanoparticles

**DOI:** 10.3390/ma18030580

**Published:** 2025-01-27

**Authors:** Jissell Alvarez-Sandoval, Gloria A. Guillen Melendez, Raymundo A. Pérez-Hernández, Joel H. Elizondo-Luevano, Rocío Castro-Ríos, Miroslava Kačániová, Carlos R. Montes de Oca-Saucedo, Adolfo Soto-Domínguez, Abelardo Chávez-Montes

**Affiliations:** 1 Departamento de Química, Facultad de Ciencias Biológicas, Universidad Autónoma de Nuevo León, San Nicolás de los Garza C.P. 64455, NL, Mexico; jissell.alvarezsnd@uanl.edu.mx (J.A.-S.); raymundo.perezhrz@uanl.edu.mx (R.A.P.-H.); joel.elizondolv@uanl.edu.mx (J.H.E.-L.); 2 Departamento de Histología, Facultad de Medicina, Universidad Autónoma de Nuevo León, Monterrey C.P. 64460, NL, Mexico; gloria.guillenmln@uanl.edu.mx (G.A.G.M.); carlos.montess@uanl.edu.mx (C.R.M.d.O.-S.); 3 Laboratorio de Ciencias Naturales, Facultad de Agronomía, Universidad Autónoma de Nuevo León, General Escobedo C.P. 66050, NL, Mexico; 4 Departamento de Química Analítica, Facultad de Medicina, Universidad Autónoma de Nuevo León, Monterrey C.P. 64460, NL, Mexico; rocio.castrors@uanl.edu.mx; 5 School of Medical & Health Sciences, University of Economics and Human Sciences in Warsaw, Okopowa 59, 01 043 Warszawa, Poland; m.kcaniova@vizja.pl; 6 Institute of Horticulture, Faculty of Horticulture and Landscape Engineering, Slovak University of Agriculture, Tr. A. Hlinku 2, 94976 Nitra, Slovakia

**Keywords:** Asteraceae, HeLa cells, HaCaT cells, PLGA, nanoparticles, plant extract, hemolysis, antiproliferative effect, *Vernonia greggii*, LC-MS

## Abstract

*Vernonia greggii* belongs to the Asteraceae family, and some members of this family have been reported to possess anticancer properties. This study evaluated the antiproliferative effect of *V. greggii* methanol extract (ME), both in its free form and encapsulated into poly(lactic-co-glycolide) (PLGA) nanoparticles (NPs), on human cervical cancer cells (HeLa) and human epidermal keratinocytes (HaCaT). The extract was subsequently sub-fractionated into n-hexane (F-He), methanol (F-Me), and distilled water (F-Ac) fractions, and their antiproliferative effects were assessed. Time-dependent toxicity on HeLa cells was observed for the free-form fractions, with the F-Me fraction showing the highest efficacy compared to the others. Additionally, an NP formulation based on PLGA and F-Me (NPs F-Me) was developed, achieving 64.21% encapsulation efficiency and 11.38% drug loading. The NPs had an average size of 146.9 nm, a polydispersity index (PDI) of 0.103, and a ζ-potential of 23.3 mV. NPs F-Me were tested on HeLa and HaCaT cells, with toxicity observed at concentrations of 300 and 500 μg/mL, affecting tumor cell morphology. Furthermore, the hemolytic activity of F-Me and NPs F-Me was evaluated. The major bioactive compounds in the F-Me fraction were identified using Liquid Chromatography–Mass Spectrometry (LC-MS). These findings suggest that the F-Me fraction of *V. greggii* exerts an antineoplastic effect both in its free form and when encapsulated in nanoparticles.

## 1. Introduction

Cancer is a major concern of the 21st century, with every fourth person facing a lifetime risk. It is characterized by the unchecked proliferation of abnormal cells originating from specific organs or systems in the body [1]. According to GLOBOCAN 2022, there were 19,976,499 new cancer cases and 9,743,832 deaths globally, with cervical cancer contributing 662,301 new cases and 348,874 deaths [2,3].

In Mexico, the National Institute of Statistics and Geography (INEGI) reported 1,122,249 deaths in 2021, of which 90,123 were attributed to malignant tumors [4,5]. Among women aged 20 to 29, malignant cervical tumors had the highest mortality rate, with 0.10 deaths per 10,000 inhabitants, followed closely by leukemia-related deaths [6].

Despite the availability of treatments, cervical cancer remains a significant global and national health issue [7,8], not only leading to high mortality rates but also deteriorating patients’ quality of life due to treatment side effects and the potential for resistance in recurrent cases.

While conventional medicine has achieved significant advancements [9,10], traditional medicine continues to be widely utilized in various regions [11,12]. Its accessibility often makes it the preferred option for treating illnesses and ailments in many communities [13,14,15].

Mexico is home to over 8000 species of vascular plants with medicinal uses [13,14]. The Asteraceae family, widely distributed globally, includes 417 genera and 3050 native species in Mexico, 1988 of which are endemic [16]. Members of this family are known for their broad range of therapeutic properties, including anti-inflammatory and antioxidant capabilities [17]. The genus *Vernonia*, part of this family, contains over 1500 species [18].

Various studies have shown that the main compounds found in this genus include flavonoids, phenolic acids, acetylenes, sesquiterpene lactones, terpenes, alkaloids, triterpenes, and lignans. Sesquiterpene lactones, in particular, serve as chemotaxonomic markers for this genus and have demonstrated biological activities such as antifungal, insecticidal, cytotoxic, and antitumor properties [17,19], the latter being especially relevant to this study.

Nanomedicine has gained importance in the medical field due to its use of nanoscale delivery systems, offering alternatives for treating diseases like cancer. These systems owe their efficacy to their physicochemical properties, including particle size, uniformity, and surface charge. Additionally, various biodegradable materials can be used to develop these systems [20,21].

Poly(lactic-co-glycolide)(PLGA) is a biodegradable and biocompatible polymer extensively studied for drug delivery. Nanoparticles (NPs) made from PLGA typically range in size from 10 to 1000 nm [22,23]. These delivery systems offer several advantages, such as controlled release of the active ingredient and the ability to modify their structure for enhanced targeting of specific cells or organs [24].

Due to these qualities, PLGA is considered one of the most suitable copolymers for formulating new pharmaceutical therapies [25]. Furthermore, this polymer is approved by the U.S. Food and Drug Administration (FDA) and the European Medicines Agency (EMA) for various biomedical applications. As of 2022, the FDA had approved around 25 pharmaceutical formulations based on PLGA [26].

Several studies have demonstrated the potential of nanoparticles for delivering natural bioactives such as flavonoids, alkaloids, plant extracts, and by-products [27,28]. For instance, PLGA nanoparticles loaded with doxorubicin (DOX) exhibited an IC_50_ of 100 ng/mL on murine fibrosarcoma cells (L929) [29]. DOX-NPs were also effective against esophageal squamous cell carcinoma (KYSE30), with an IC_50_ of 40–60 µg/mL [30].

Similarly, the extract of *Hypericum perforatum* encapsulated in PLGA-based NPs significantly inhibited the viability of KYSE30 cells at concentrations of 0.6–0.7 mg/mL, with a marked decrease in cyclin D1 expression [30]. In 2020, Ibrahim, W.N. et al., reported the effect of a formulation of NPs using PLGA as a biocompatible coating material loaded with thymoquinone—the main compound of the traditional herb *Nigella sativa*, which has wide therapeutic applications and recognized anticancer properties—on human melanoma cells (A375) [31]. Additionally, nanoparticles developed using a mixture of PLGA and poly(ε-caprolactone) (PCL), loaded with essential oil (EO) from *Boswellia sacra* oleoresin, enhanced apoptosis in human breast adenocarcinoma cells (MCF-7) while showing controlled release over 72 h, further supporting the potential of PLGA-based NPs in cancer treatment [32].

The main objective of this research was to determine the antineoplastic activity of the methanolic extract of *Vernonia greggii*, evaluated in vitro both in its free form and encapsulated in PLGA nanoparticles, on cervical cancer cells (HeLa). Additionally, its selectivity index on human epidermal keratinocytes (HaCaT) and its toxic effects on human erythrocytes were assessed. The study also explores the formulation of PLGA-based nanoparticles as a strategy to improve the bioavailability of the bioactive compounds present in the extract and optimize their potential in oncological therapies. The bioactive compounds of the extract were identified using Liquid Chromatography–Mass Spectrometry (LC-MS), aiming to contribute to the design of advanced biomaterials applicable in pharmacological treatments, natural therapies, and controlled drug release systems.

## 2. Materials and Methods

### 2.1. Chemicals and Cell Lines

All solvents and chemicals used were of analytical grade and purchased from CTR Scientific^®^ (https://ctrscientific.com/; accessed on 25 September 2024), located in Monterrey, NL, Mexico.

The human cervical cancer cell line (HeLa, ATCC CCL-2) and human epidermal keratinocytes (HaCaT) were obtained from the American Type Culture Collection (ATCC, Manassas, VA, USA). Human erythrocytes were kindly provided by the Department of Histology, Faculty of Medicine, Universidad Autónoma de Nuevo León (UANL), Monterrey, Mexico. Ethical guidelines for their use are detailed in the corresponding section.

### 2.2. Plant Material

The plant was collected from Sierra Madre Oriental, NL, Mexico (25°22′00″ N, 100°33′00″ W/25.36666667, −100.55) in January 2023. The plant material was identified as *Vernonia greggii* (Herbarium ID No. 25590) by the curator of the Herbarium at Facultad de Ciencias Biológicas (FCB), UANL. Botanical names and plant families were taxonomically validated using The Plant List (http://www.theplantlist.org; accessed on 25 September 2024) and the International Plant Names Index (IPNI) (https://www.ipni.org/; accessed on 25 September 2024).

### 2.3. Plant Extraction

The aerial parts of the plant (flowers, leaves, and stems) were thoroughly dried and ground using a mechanical manual mill. The methanolic extract (ME) was prepared through maceration with methyl alcohol (MeOH) [33]. In this process, 100 g of dried plant material was placed in a 1000 mL flask, and 500 mL of absolute MeOH were added. The mixture was left to stand for 72 h, with the solvent replaced every 24 h [34]. The extract was then filtered and evaporated under reduced pressure at 28 °C and 100 rpm using a rotary evaporator. Finally, the extract was dried for 48 h, protected from light to prevent photodegradation, and stored in amber vials at 4 °C until further use [35]. After obtaining the crude methanolic extract (ME), it was partitioned using solvents of increasing polarity, including n-hexane, methanol, and distilled water. This process yielded three distinct fractions: hexanic (F-He), methanolic (F-Me), and aqueous (F-Aq) [36]. The extraction yields of the crude extract (ME) and fractions (F-He, F-Me, and F-Aq) were calculated using the following Equation (1):

(1) $$Yield\;(\%)=\frac{Final\;weight}{Initial\;weight}\times 100$$

### 2.4. Preliminary Phytochemical Screen

The crude extract (ME) and its fractions underwent a phytochemical screening process to identify the presence of various secondary metabolites. Tests were conducted to detect alkaloids (Dragendorff), carbohydrates (anthrone), coumarins (sodium hydroxide), flavonoids (Shinoda), quinones (Bornträger), saponins (sodium bicarbonate), sesquiterpene lactones (Baljet), sterols and triterpenes (Liebermann–Burchard), and tannins (ferric chloride) [37,38]. Results were reported as presence (+) or absence (−) for each class of compounds.

### 2.5. Chromatographic Analysis

The methanolic fraction (F-Me) was analyzed using a 2695 HPLC system (Waters, Milford, MA, USA) equipped with a 2696 PDA detector to obtain the UV–Vis spectra of its peaks. Further analysis of the F-Me was performed using a UHPLC Ultimate 3000 system equipped with a VWD-3400RS variable wavelength UV–Vis detector (Dionex Thermo Scientific, Sunnyvale, CA, USA) and coupled to an ion-trap LCQ Fleet Mass Spectrometry detector (Thermo Finnigan, San Jose, CA, USA).

Chromatographic separation was carried out using a Discovery HS F5 column (15 cm × 2.1 mm, 3 µm; Supelco, Torrance, CA, USA) with mobile phases consisting of (A) aqueous 0.1% formic acid and (B) acetonitrile containing 0.1% formic acid, at a flow rate of 0.25 mL/min. The gradient started with 10% B for 1 min, followed by a linear increase to 70% B over 19 min. After 20 min, the system was returned to initial conditions with a 5 min gradient, and an equilibration period of 15 min was included between injections. The column oven temperature was set at 45 °C, with an injection volume of 5 µL. UV–Vis spectra were recorded from 200 to 600 nm. LC-MS analyses were conducted using an electrospray ionization (ESI) source operating in both positive and negative ionization modes. For both modes, the capillary temperature was set at 300 °C, the sheath gas flow rate was 70 arbitrary units, and the auxiliary gas flow rate was 20 arbitrary units. Additional conditions included a capillary voltage of 10 V and a lens tube voltage of 60 V in the positive ionization mode, while for the negative mode, the capillary voltage was set to −36 V and the tube lens voltage to −110 V. Mass spectrometry (MS) and tandem mass spectrometry (MS^2^) experiments were performed to characterize the F-Me extracts. These analyses included full-scan acquisition within a mass-to-charge ratio (*m*/*z*) range of 50 to 2000, as well as data-dependent analysis of the most intense ions or specific target ions. Collision-induced dissociation (CID) was employed using a collision energy of 35%.

### 2.6. Development of the NPs

Nanoparticles (NPs) were prepared using the nanoprecipitation method as described by Elizondo-Luevano et al. (2023) [39]. Two organic phases (OP1 and OP2) and one aqueous phase (AP) were used. OP1 consisted of 12 mL of acetone with 60 mg of PLGA 75:25 (Resomer^®^ RG 755 S, poly(lactic-co-glycolide), molecular weight ~61.1 kDa; Sigma-Aldrich^®^, St. Louis, MO, USA) dissolved. OP2 contained 12 mg of F-Me dissolved in 2 mL of acetone and filtered through Whatman filer paper (42.5 mm). OP1 and OP2 were mixed, sonicated for 5 min (Ultrasonic Branson 2510MT, Merck KGaA, Darmstadt, Germany), and injected into 12 mL of distilled water (AP). The mixture was evaporated under reduced pressure using a rotary evaporator (Laborota 4003, Heidolph Instruments, Schwabach, Germany) for 15 min at 1000 rpm at room temperature. A nanoparticle control (NPs without F-Me) was prepared using the same procedure. The nanoformulation containing F-Me was referred to as “NPs F-Me”. The PLGA nanoparticles (PLGA NPs) prepared without F-Me were used as controls and referred to as NPs control.

### 2.7. Characterization of the NPs

As part of the characterization of the nanoparticles (NPs), particle size (in nanometers, nm), polydispersity index (PDI), and zeta potential (ζ) were measured to evaluate the surface charge density. Nanoparticles with a ζ potential between −10 and +10 mV were classified as “neutral”, whereas those with ζ potential greater than +30 mV or less than −30 mV were categorized as “strongly cationic” or “strongly anionic”, respectively [40]. The average size, PDI, and ζ potential of the NPs were determined using photon correlation spectroscopy (PCS) with a Zetasizer Nano-Zs90 (Malvern Instruments, Worcestershire, UK). Measurements were performed after dispersing the NPs in 1 mL of distilled water [27].

#### Encapsulation Efficiency

To measure the extract immobilized in the NPs, the formulations were centrifuged at 25,000 rpm at 4 °C for 4 h. The encapsulated extract within the NPs and the non-encapsulated extract in the supernatants were quantified. Quantitative analysis of the extract was performed using a calibration curve prepared with the extract and analyzed on a GENESYS 10S UV–Vis spectrophotometer (Thermo Scientific, Waltham, MA, USA). The encapsulation efficiency (*ee* %) and drug load (*dl* %) were calculated based on Azzazy H.M.E. et al. (2022) [32], with modifications, as follows (2) and (3):

(2) $$ee\;\%=\frac{{EX}_{I}}{{EX}_{E}}\times 100$$

where *EX_I_* corresponds to the mass of the initial extract and *EX_E_* corresponds to the mass of the encapsulated extract.

(3) $$dl\;\%=\frac{M_{N}}{M_{E}}\times 100$$

where *M_N_* corresponds to the total mass of the nanoparticles and *M_E_* corresponds to the mass of the nano-encapsulated extract.

### 2.8. Cytotoxicity Assay

The cytotoxic effects of the extracts and fractions of *Vernonia greggii* were assessed using the MTT assay (3-(4,5-dimethylthiazol-2-yl)-2,5-diphenyltetrazolium bromide) [41]. All treatments were prepared in Dulbecco’s Modified Eagle Medium (DMEM, Gibco™, Grand Island, NY, USA), with the negative control consisting of DMEM without additives. The methanolic extract (ME) of *V. greggii* was evaluated at concentrations of 0, 100, 200, 300, 400, and 500 μg/mL, while the fractions and nanoparticles (NPs) were tested at concentrations of 0, 10, 50, 100, 300, and 500 μg/mL. All treatments were sterilized by filtration using Whatman™ UNIFLO™ 0.45 μm PES filters (Cytiva, Global Life Sciences Solutions USA LLC, Marlborough, MA, USA).

The cytotoxic activity was evaluated in HeLa cervical cancer cells and HaCat keratinocytes, both of which are adherent cell lines. Cells were seeded in 96-well, clear, flat-bottom microplates and incubated under controlled conditions at 37 °C in an atmosphere of 5% CO_2_ and 95% air. Cell viability and culture conditions were performed according to Guillén-Meléndez et al. (2021) [42]. After 24 h of incubation, the percentage of cell viability (*CV*) was determined using the following Formula (4) [36]:

(4) $$CV\;\%=\frac{\;{Abs}_{570nm}Treatment}{\;{Abs}_{570nm}\;Negative\;control}\times 100$$

The selectivity index (*SI*) was calculated as follows (5) [36]:

(5) $$SI=\frac{{IC}_{50}\;Normal\;cells}{{IC}_{50}\;Tumor\;cells}$$

The *IC*₅₀ values were calculated for each treatment. Extracts were classified based on their cytotoxicity as highly cytotoxic (*IC*₅₀ < 10 μg/mL), cytotoxic (*IC*₅₀ > 10 and <100 μg/mL), moderately cytotoxic (*IC*₅₀ > 100 and <1000 μg/mL), or potentially non-cytotoxic (*IC*₅₀ > 1000 μg/mL) [43].

### 2.9. Nucleus Contrast DAPI Assay

The DAPI (4,6-diamidino-2-phenylindole; Thermo Fisher Scientific Inc., Waltham, MA, USA) assay was used to evaluate nuclear morphological changes induced by plant extract treatments and to further validate the findings from the MTT assay, as previously described [44]. Morphometric analysis of the images was performed using ImageJ^®^ software (v1.51; National Institutes of Health, Bethesda, MD, USA) to calculate the percentage of area covered per well.

### 2.10. Hemolytic Assay

The hemolytic activity on human erythrocytes was assessed using a previously described hemolysis assay [39,45], employing a 5% erythrocyte suspension. Treatment concentrations ranging from 100 to 1000 µg/mL were evaluated. Each treatment was prepared in 2 mL tubes, where a 5% erythrocyte suspension was added. Distilled water and PBS served as positive and negative controls, respectively. The samples were incubated for 35 min at 37 °C, followed by centrifugation at 4 °C for 5 min at 13,000 rpm. Subsequently, 200 µL of the supernatant from each sample was transferred to a 96-well transparent microplate with a concave bottom. The optical density (OD) of the released hemoglobin was measured at 550 nm using a microplate reader. The percentage of hemolysis for each sample was calculated using the following Formula (6):

(6) $$\%\;Hemolysis=\frac{{DO}_{540}\;Treatment-{DO}_{540}\;Negative\;control}{{DO}_{540}\;Positive\;control-{DO}_{540}\;Negative\;control}\times \;100$$

### 2.11. Statistics

All data are presented as means ± standard deviation (SD) from at least three independent experiments performed in triplicate. A one-way analysis of variance (ANOVA) was used to evaluate significant differences among the tested concentrations. Dunnett’s post hoc test was applied to compare the cytotoxicity of the treatments with their respective controls, while Tukey’s post hoc test was used to assess differences among treatment means. Statistical analyses were performed using GraphPad Prism 9 software (GraphPad Software Inc., San Diego, CA, USA). IC₅₀ values (half-maximal inhibitory concentration) were calculated using the Probit test with the Quest Graph™ IC₅₀ Calculator tool (AAT Bioquest, Inc., Pleasanton, CA, USA).

## 3. Results

### 3.1. Extraction Yields and Phytochemical Analysis

The extraction yields obtained from the maceration of the crude methanolic extract and its hexane, methanolic, and aqueous fractions are presented in Table 1. The proportions of these fractions within the crude methanolic extract were calculated as follows: 0.34% *w*/*w* for the hexane fraction and 16.6% *w*/*w* for the aqueous fraction. Colorimetric phytochemical tests identified a wide range of bioactive/phytoactive components, including flavonoids, sterols, triterpenes, sesquiterpene lactones, tannins, carbohydrates, and coumarins (Table 1).

### 3.2. Chromatographic Analysis

**Figure 1:**
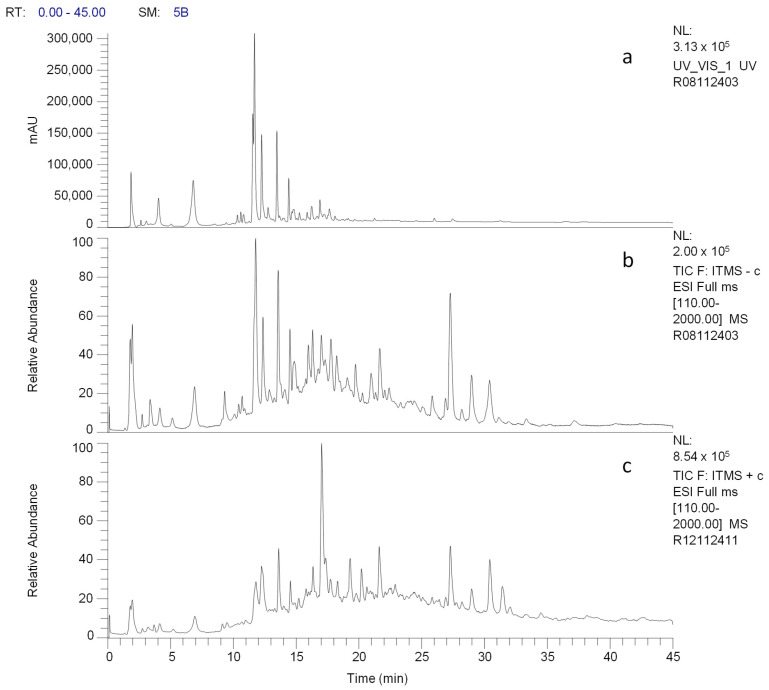
Chromatograms of the F-Me methanolic extract (0.1 mg/mL) obtained using a Discovery HS F5 column under the conditions described in Section 2.5. (**a**) UV–Vis at 254 nm. (**b**) Total ion current (TIC) in ESI-negative mode. (**c**) TIC in ESI-positive mode.

Several reversed-phase and HILIC HPLC columns operating under different conditions were tested for F-Me extracts, and their performance was evaluated using PDA UV–Vis chromatograms at wavelengths of 210, 254, 280, 310, 350, 450, and 550 nm. The Discovery HS F5 chromatographic system, described in Section 2.5, was selected based on its ability to generate the highest number of chromatographic signals with adequate resolution using a wavelength of 254 nm. This system was subsequently integrated into the UHPLC-ESI/MS setup, and chromatograms were obtained in both positive and negative ionization modes. Figure 1 presents the UV–Vis and MS total ion current (TIC) chromatograms for an F-Me methanolic solution (0.1 mg/mL). A summary of the UV–Vis and MS data for the identified compounds is provided in Table 2.

### 3.3. Cytotoxic Activity of V. greggii Extract and V. greggii Fractions

**Figure 2:**
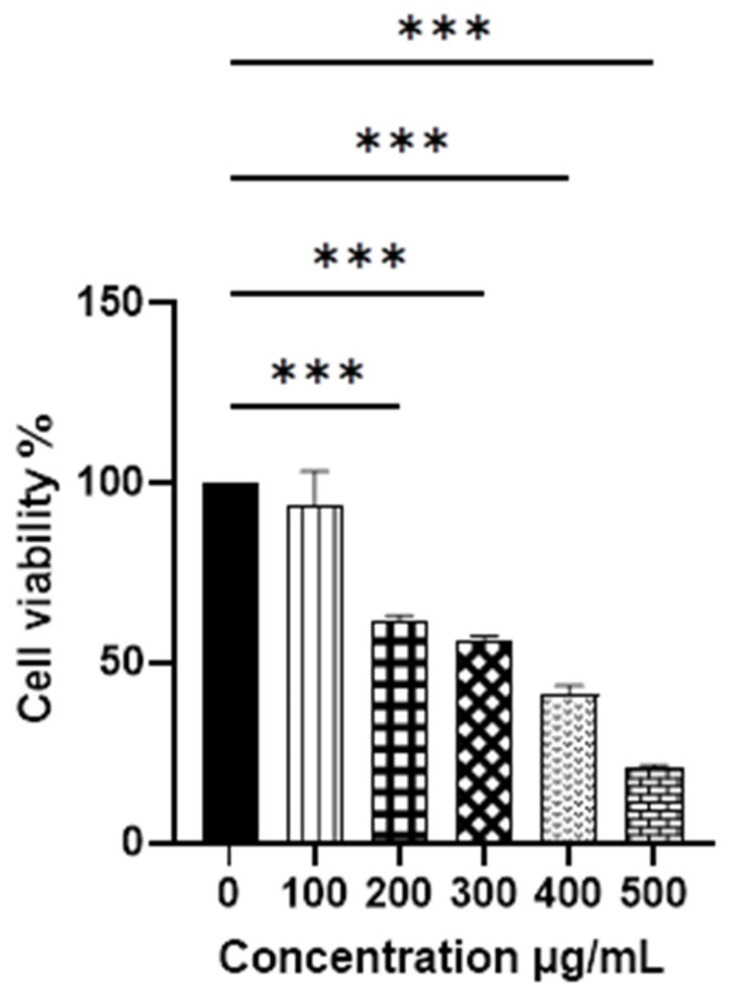
MTT assay on HeLa cell cultures. The cytotoxic effect of unencapsulated methanolic extract (ME) (100, 200, 300, 400, and 500 μg/mL) compared to the negative control (0 μg/mL) at 24 h is shown. Data are presented as mean ± standard deviation (SD). ANOVA was performed with Dunnett’s post hoc test for the comparison of means (*** *p* < 0.001).

The MTT assay was used to evaluate the cytotoxic activity of *V. greggii* at different concentrations, with results measured 24 h post-treatment. Figure 2 illustrates the cytotoxic effect of the crude *V. greggii* methanolic extract (ME) at concentrations ranging from 100 to 500 μg/mL on HeLa tumor cells. A significant decrease in cell viability (CV) to 61.27% was observed at 200 μg/mL. To date, there are no reports in the literature documenting the anticancer activity of *V. greggii* against this tumor line.

**Figure 3:**
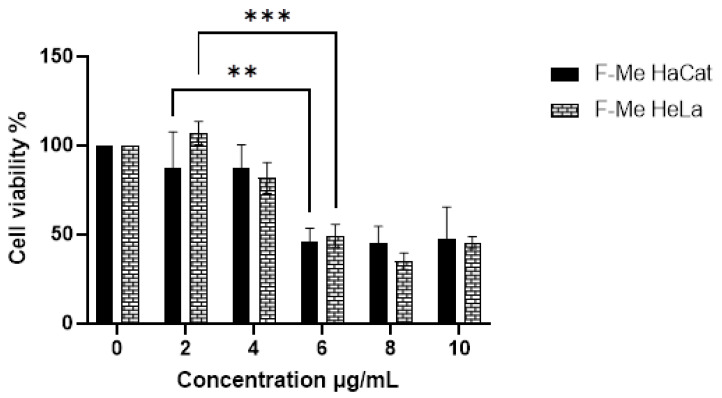
MTT assay on HaCat and HeLa cell cultures. The cytotoxic effect of unencapsulated F-Me (2, 4, 6, 8, and 10 μg/mL) compared to the negative control (0 μg/mL) at 24 h is shown. Data are presented as mean ± SD. ANOVA was performed with Tukey’s post hoc test for the comparison of means (** *p* < 0.01, and *** *p* < 0.001).

**Figure 4:**
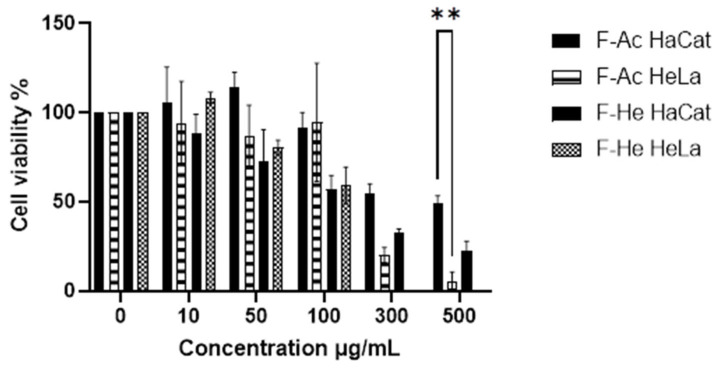
MTT assay on HaCat and HeLa cell cultures. The cytotoxic effect of unencapsulated F-Ac and F-He (10, 50, 100, 300, and 500 μg/mL) compared to the negative control (0 μg/mL) at 24 h is shown. Data are presented as mean ± standard deviation (SD). ANOVA was performed with Tukey’s post hoc test for the comparison of means (** *p* < 0.01).

Subsequently, the cytotoxic effect of the fractions was examined. The methanolic fraction (F-Me) demonstrated cytotoxic activity at concentrations as low as 6 μg/mL, reducing CV to 49.17% and 45.75% in HeLa and HaCat cell cultures, respectively (Figure 3). The hexane fraction (F-He) exhibited a CV of 57% and 59% in HaCat and HeLa cell cultures, respectively, at 100 μg/mL. The aqueous fraction (F-Aq), at a concentration of 300 μg/mL, reduced CV to 54% and 29% in HaCat and HeLa cells, respectively, as shown in Figure 4. The corresponding IC_50_ values and selectivity indices (SI) are summarized in Table 3. The SI values were below three, indicating that the treatments lacked high selectivity [46].

### 3.4. Polymeric Nanoparticle Development

Based on the cytotoxicity results, the methanolic F-Me fraction, which exhibited the highest cytotoxic effect, was selected for nanoencapsulation. The physical characteristics of the PLGA 75:25-based nanoparticles are summarized in Table 4. The average size of NPs F-Me was 146.9 ± 1.159 nm, with a polydispersity index (PDI) of 0.103 and a zeta potential (ζ) of −23.3 mV. The nanoprecipitation procedure demonstrated high efficiency, yielding an encapsulation efficiency (ee %) of 64.21% and a drug loading (dl %) of 11.38%.

### 3.5. Cytotoxic Activity of NPs

**Figure 5:**
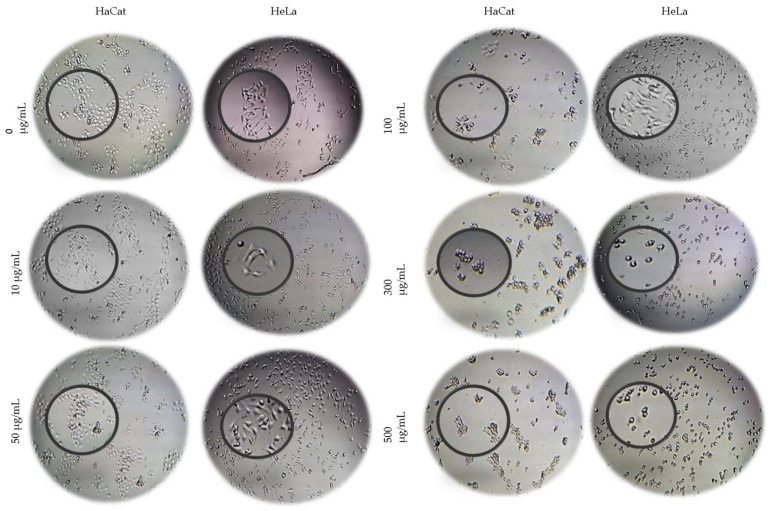
Micrographs at 4× magnification of HaCat and HeLa cells after 24 h exposure to NPs F-Me (10, 50, 100, 300, and 500 μg/mL), alongside the negative control (0 μg/mL). Morphological alterations in both cell lines were evident at higher doses (300 and 500 μg/mL).

After determining the cytotoxicity of the free fractions and preparing the nanoparticle formulations (NPs F-Me and PLGA NPs), their effects were evaluated on HaCat and HeLa cells. Using optical microscopy, morphological changes were observed in both cell lines prior to the addition of the MTT solution. These changes were evident at concentrations of 300 and 500 μg/mL after 24 h of NPs F-Me treatment. Notably, HeLa cells exhibited a more rounded morphology, likely due to a loss of cellular adhesion, which resulted in reduced cell confluence (Figure 5).

**Figure 6:**
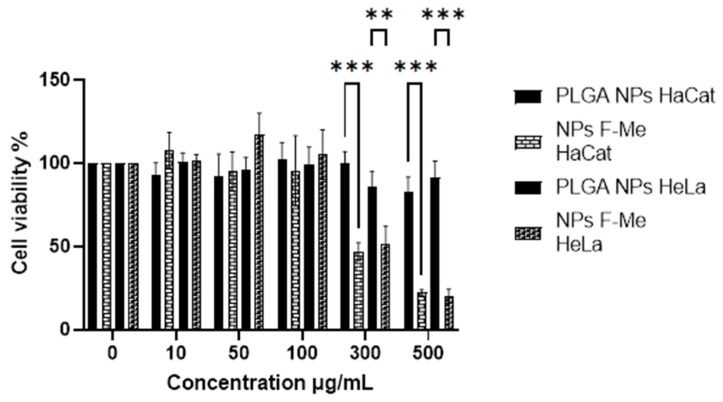
MTT assay on HaCat and HeLa cells. The cytotoxic effect of the treatments (evaluated at 24 h): PLGA NPs (unloaded NPs) and NPs F-Me (F-Me-loaded NPs)—both at concentrations of 10, 50, 100, 300, and 500 μg/mL—and the negative control (0 μg/mL). The data are presented as mean ± SD. A one-way ANOVA was performed with Tukey’s test for mean comparison (** *p* < 0.01, and *** *p* < 0.001).

The methanolic fraction of *Vernonia greggii* (F-Me), in both its free form and encapsulated within nanoparticles (NPs F-Me), exhibited antineoplastic activity against HeLa cells. This fraction demonstrated higher cytotoxicity compared to the crude extract, as well as to the n-hexane (F-He) and aqueous (F-Ac) fractions. The NPs F-Me treatment showed a dose-dependent response, with cytotoxic effects becoming more pronounced at 300 and 500 μg/mL, reducing cell viability (CV) in HeLa cells to 51.20% and 20.48%, respectively. Similarly, the treatment reduced HaCat cell viability to 47.01% and 22.58% at 300 and 500 μg/mL, respectively (Figure 6). However, the NPs F-Me did not exhibit high selectivity indices (Table 5).

### 3.6. DAPI Assay

**Figure 7:**
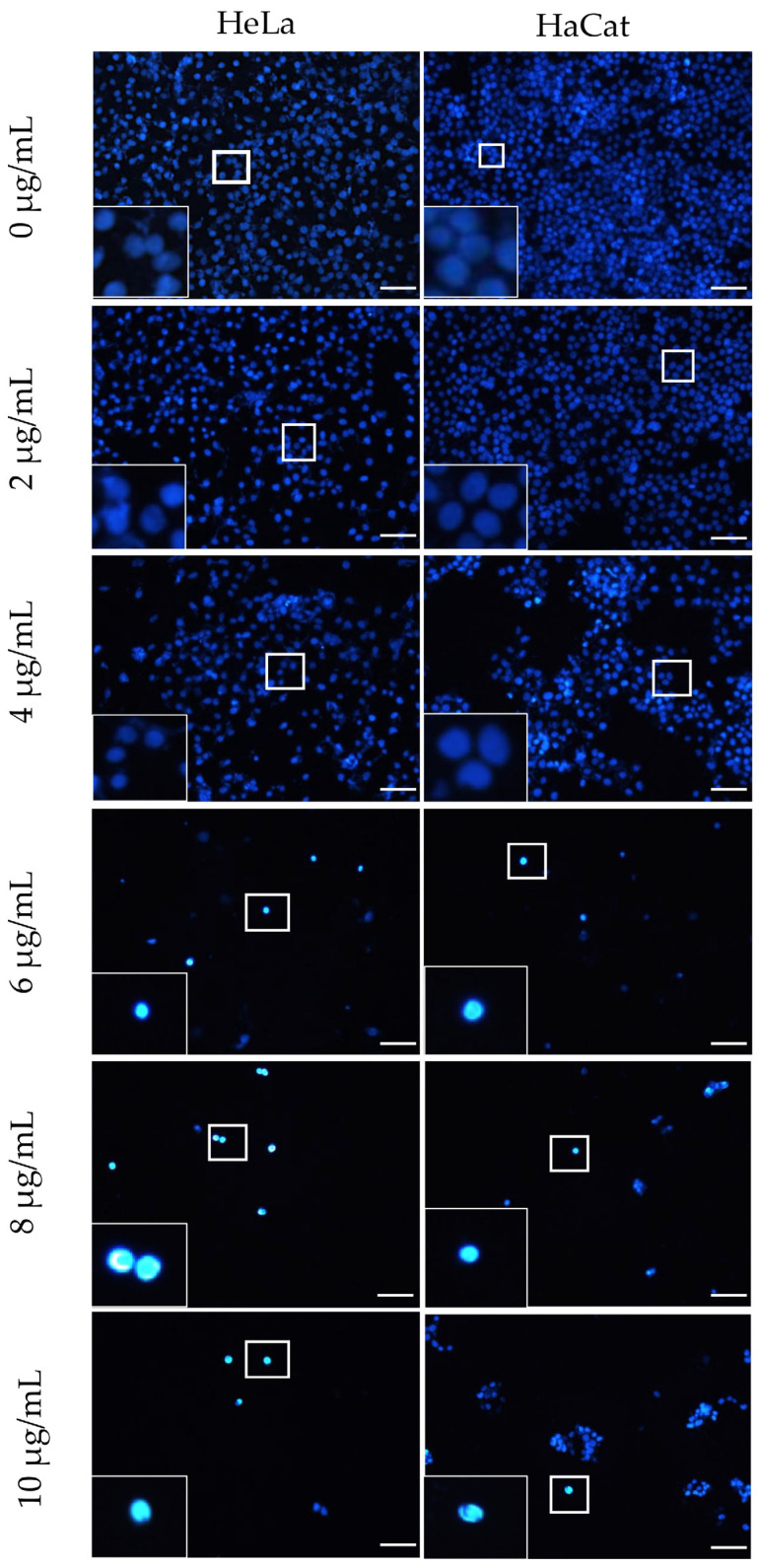
Nuclei labeling with DAPI. Micrographs of HeLa and HaCat cell cultures exposed to F-Me at concentrations of 0, 2, 4, 6, 8, and 10 μg/mL for 24 h. Morphological alterations observed in the nuclei include pyknosis, rounding, and increased fluorescence intensity. A marked decrease in cell confluence is evident starting at 4 μg/mL. Scale bar: 50 μm.

**Figure 8:**
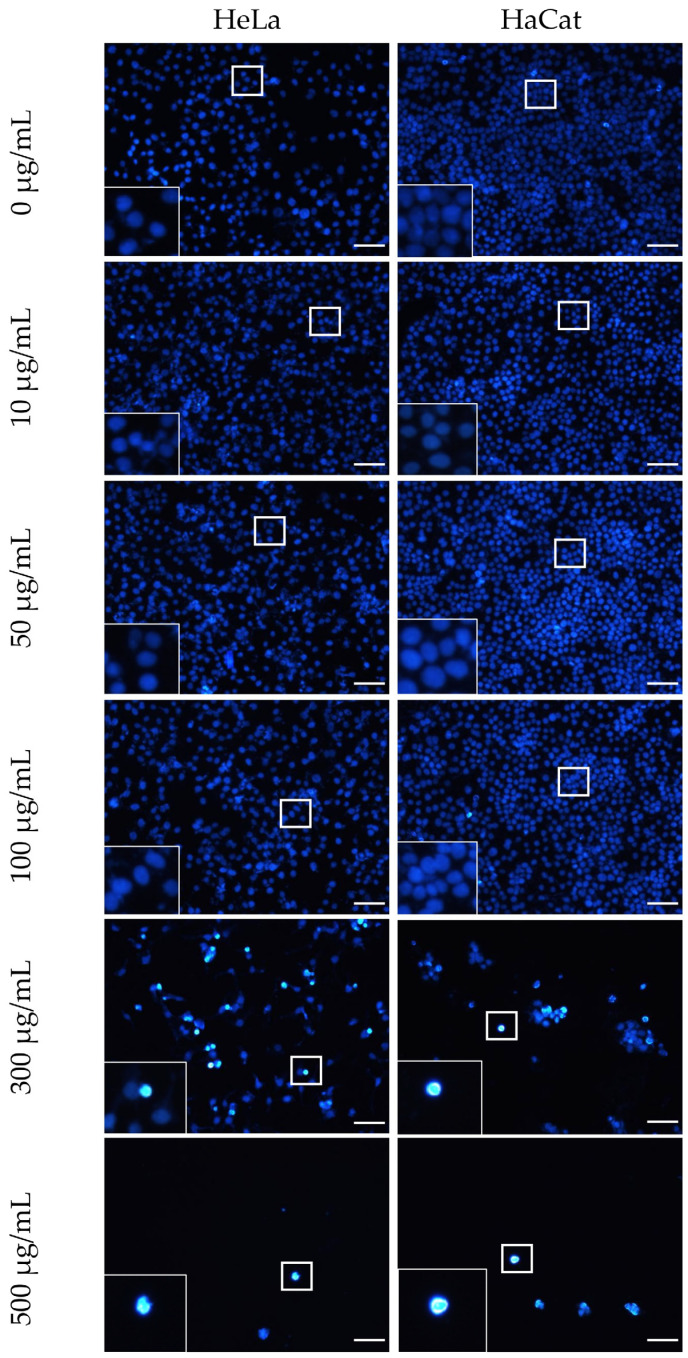
Micrographs of HeLa and HaCat cells exposed to NPs F-Me at concentrations of 0, 10, 50, 100, 300, and 500 μg/mL for 24 h. Nuclear staining was performed using DAPI. Morphological alterations in the nuclei, including pyknosis, rounding, and increased fluorescence intensity, were observed. A marked decrease in confluence was evident starting at 300 μg/mL. Scale bar: 50 μm.

**Figure 9:**
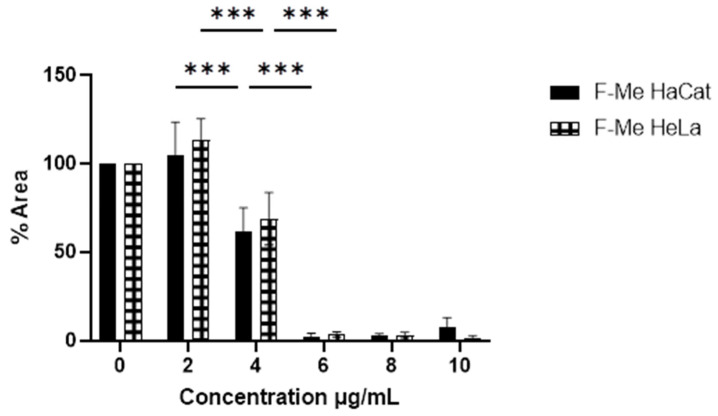
DAPI assay on HaCat and HeLa cells. The cytotoxic effect of F-Me at concentrations of 2, 4, 6, 8, and 10 μg/mL, as well as the negative control (0 μg/mL), was evaluated after 24 h. The data are presented as mean ± SD. A one-way ANOVA was performed with Tukey’s test for mean comparison (*** *p* < 0.001).

**Figure 10:**
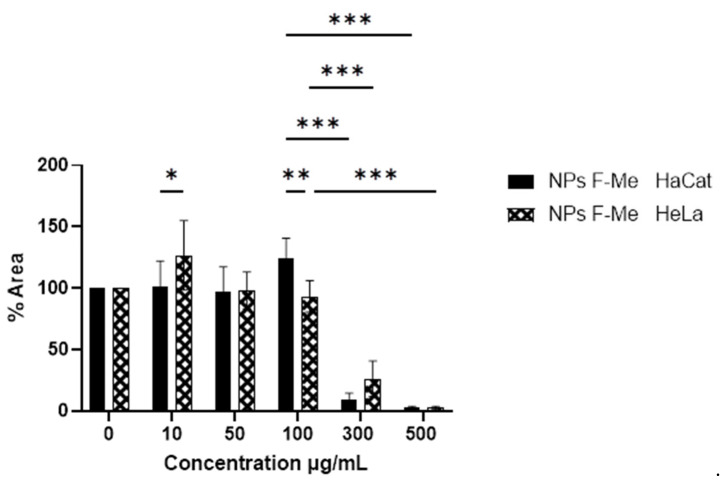
DAPI assay on HaCat and HeLa cells. The cytotoxic effect of NPs F-Me (F-Me-loaded NPs) at concentrations of 10, 50, 100, 300, and 500 μg/mL, as well as the negative control (0 μg/mL), was evaluated at 24 h. The data are presented as mean ± SD. A one-way ANOVA was performed with Tukey’s test for comparison of means (* *p* < 0.05, ** *p* < 0.01, and *** *p* < 0.001).

The toxicity of the F-Me and NPs F-Me treatments was further evaluated at the nuclear level using the DAPI assay. Cell viability was found to be inversely proportional to the percentage of cell adhesion area. Tumor cells exhibited morphological nuclear changes characteristic of cell death, including chromatin condensation, rounding, size reduction, and increased fluorescence intensity, as shown in Figure 7 and Figure 8. Quantitative analysis revealed that the F-Me treatment significantly reduced the adhesion area percentage starting at 4 μg/mL, while NPs F-Me demonstrated toxicity beginning at 300 μg/mL. These findings are presented in Figure 9 and Figure 10.

### 3.7. Hemolytic Activity

The hemolytic activity of *V. greggii* was evaluated to determine its toxicity on human erythrocytes. Both free F-Me and NPs F-Me treatments were tested, and the percentage of hemolysis is presented in Table 6. NPs F-Me exhibited hemolytic activity at concentrations of 1000 μg/mL and 800 μg/mL, with hemolysis percentages of 32.23% and 14.18%, respectively. In contrast, free F-Me demonstrated no hemolytic activity. According to the hemolysis percentage criteria established by Mesdaghinia et al. (2019), hemolysis is classified as non-hemolytic (<2%), slightly hemolytic (2–5%), or hemolytic (>5%) [47].

## 4. Discussion

Based on the results obtained, flavonoids were identified as the most abundant compounds in the methanolic extract of *V. greggii*, followed by sesquiterpene lactones, according to the preliminary phytochemical profile. Sesquiterpene lactones in the *Vernonia* genus are considered chemotaxonomic markers due to their significant biological activity, particularly their cytotoxic and antitumor properties [17]. As shown in Figure 1, the HPLC with UV–Vis and MS chromatograms revealed more than 30 chromatographic signals. A tentative identification of the compounds in the F-Me extract was made using the spectral data (λmax, MS, and MSⁿ positive and negative ions), with the results presented in Table 1. A series of mono- and di-caffeoylquinic acid derivatives were observed, corresponding to some of the most intense chromatographic signals. Additionally, several quercetin glycosides, isoquercitrin, rutin, and vixetin, were identified.

In 2022, Pakpisutkul investigated the cytotoxic effects of ethanolic and aqueous extracts from the aerial parts of *Vernonia cinerea* on colorectal cancer cell lines (SW620 and HT29). Cytotoxicity was evaluated using the MTT assay over 1, 4, and 7 days, with concentrations ranging from 100 to 700 μg/mL and 10 to 150 μg/mL for each cell line, respectively. The results demonstrated that the ethanolic extract exhibited greater cytotoxic activity compared to the aqueous extract, with IC₅₀ values below 100 μg/mL on the first day. In contrast, the aqueous extract exhibited IC₅₀ values ranging from 300 to 800 μg/mL [48]. These findings align with our study, as the cytotoxic effect of F-Me was observed at concentrations below 100 μg/mL, demonstrating greater toxicity than F-Ac, which had an IC₅₀ of 263.34 μg/mL against HeLa cells.

Other researchers evaluated the dichloromethane fraction of the ethanolic extract of *V. cinerea* for toxicity against HeLa cells, reporting IC₅₀ values of 76.92 and 59.35 μg/mL at 48 h for the ethanolic extract and fraction, respectively [49]. In contrast, our study showed IC₅₀ values of 268.08 μg/mL and 4.33 μg/mL at 24 h for the crude methanolic extract and its methanolic fraction, respectively, indicating that the F-Me fraction in this study exhibited greater toxicity against HeLa cells.

In 2016, Thomas and colleagues studied *Vernonia condensata* using the MTT and Trypan Blue assays. They observed a significant impact on cell proliferation at a concentration of 5 mg/mL. The most susceptible cancer cell lines to *V. condensata* were Reh and Nalm6 (leukemic cells), with IC₅₀ values of 10.7 and 9.0 μg/mL, respectively. In comparison, HEK293T cells (human embryonic kidney cells) were affected at an IC₅₀ of 26.0 mg/mL [50].

Our results on the behavior of the methanolic fraction of *V. greggii* nanoencapsulated in PLGA 75:25 on HaCat and HeLa cells demonstrated signs of cellular damage, with IC₅₀ values of 299.90 and 271.37 µg/mL with an SI of 0.94, respectively. Therefore, the NPs F-Me formulation showed higher toxicity compared to previously reported data for other tumor cells.

The encapsulation of the F-ME treatment resulted in a low selectivity index (SI), likely due to several factors inherent to nanoparticle-based drug delivery systems. Encapsulation can reduce drug efficacy by limiting bioavailability, altering release kinetics, or causing unfavorable interactions between the active compound and the nanoparticles. For instance, encapsulation may restrict the drug’s release at the target site, and slower or controlled release profiles can impact its therapeutic potential [51,52,53,54,55]. Studies have shown that a higher lactic acid to glycolic acid (LA/GA) ratio, combined with a higher molecular weight, increases polymer hydrophobicity, leading to slower drug release due to reduced degradation. While PGA is a hydrophilic and crystalline polymer with a rapid degradation rate under physiological conditions, PLA is hydrophobic, with slower degradation and lower mechanical resistance [51].

In this study, interactions between the active molecules and the polymer may have delayed release during the assay. The system exhibited a biphasic release profile, characterized by a moderate initial burst within the first 24 h—attributable to the extract’s hydrophobicity—followed by sustained release influenced by the high molecular weight of PLGA (76,000 Mw) [52]. The degradation of glycolic acid units creates LA-rich fragments prone to crystallization due to the LA/GA ratio (75:25) [54]. This progressive crystallization increases hydrophobicity, alters the polymer matrix structure, and hinders drug diffusion, potentially resulting in subtherapeutic doses compared to the free-form extract [54].

Also, it has been demonstrated that drug-loaded PLGA-NPs with antineoplastic agents as paclitaxel enhance their cytotoxic activity over time as the active compounds are gradually released. This sustained release allows the active ingredients to exert their effects progressively, depending on the incubation period in in vitro cell cultures [55].

To date, no reports exist on the cytotoxic activity of *V. greggii* nanoencapsulated in PLGA. Previous studies, however, have highlighted the use of a sesquiterpene lactone, vernolepin, isolated from *Vernonia lasiopus* and nanoencapsulated in polylactic acid (PLA). This formulation was evaluated solely for antiparasitic activity [56].

Information is also available on silver nanoparticles (NPs) loaded with an aqueous extract of *V. amygdalina* against the MCF-7 breast cancer cell line, reporting an IC₅₀ value of 6.11 μg/mL at 72 h. This treatment induced apoptosis and caused G1 phase cell cycle arrest [57]. Given that *V. greggii* and *V. amygdalina* belong to the same Asteraceae family, it is plausible that they share a similar mechanism of action. However, further studies are needed to confirm this hypothesis [19].

Another study evaluated the ethanolic extract of *V. amygdalina* leaves on MCF-7 and MDA-MB-231 breast cancer cell lines, determining IC₅₀ values that inhibited cell proliferation. For MCF-7 cells, IC₅₀ values of 100, 66, and 56 μg/mL were observed at 24, 48, and 72 h, respectively. For MDA-MB-231 cells, IC₅₀ values of 83, 53, and 46 μg/mL were reported for the same time points [58].

In the present study, the leaves of *V. greggii* were also used to obtain the methanolic extract. However, the cytotoxicity of the free F-Me fraction on HeLa cells was not dose- or time-dependent when compared to the ethanolic extract of *V. amygdalina*.

The cytotoxic effects of aqueous and ethanolic extracts of *Vernonia mespilifolia* were also evaluated against HeLa cells using whole plant extracts. Dual staining with Hoechst 33342/PI revealed significant cell death induced by the ethanolic extract, with an IC₅₀ value of 149.12 μg/mL. Conversely, the aqueous extract exhibited negligible cytotoxic effects, with an IC₅₀ > 200 μg/mL [59]. This suggests that both plants from the Asteraceae family exhibit cytotoxic properties. In this study, the aqueous fraction of *V. greggii* from its aerial parts showed a cytotoxic effect against cervical cancer cells with an IC₅₀ of 262.34 μg/mL, aligning with the median inhibitory concentration observed for *V. mespilifolia* [59].

This anticancer activity has been linked to secondary metabolites, particularly sesquiterpene lactones, which are known for their antitumor and antineoplastic effects. In Mohammad’s study [60], sesquiterpene lactones isolated from *Artemisia macrocephala* demonstrated growth inhibition of 63.39% and 81.25% at 500 and 1000 μg/mL, respectively, in HeLa cells. While no specific compounds were isolated in the current study, the presence of sesquiterpene lactones in the methanolic extract was confirmed. Furthermore, the fractionated methanolic, aqueous, and hexane extracts of *V. greggii* showed reduced cell viability, with the aqueous fraction exhibiting an IC₅₀ of 262.34 μg/mL. These results align with the cytotoxicity reported for sesquiterpene lactones in Mohammad’s study [60].

Previous studies have reported that caffeoylquinic acids, such as 3,4-dicaffeoylquinic acid and 1,5-dicaffeoylquinic acid, exhibit antioxidant, cytotoxic, DNA-protective, neuroprotective, and hepatoprotective properties. Additionally, these compounds have been shown to exert cytotoxicity through apoptosis induction and demonstrate inhibitory effects on α-glucosidase activity [61,62,63]. Moreover, 3,4-dicaffeoylquinic acid possesses anti-influenza viral activity, enhancing viral clearance by increasing the expression of tumor necrosis factor-related apoptosis-inducing ligand (TRAIL) [64].

PLGA-based NPs are widely used to encapsulate conventional drugs and plant extracts. As previously discussed, nanosystems can target tumor tissues through the enhanced permeability and retention (EPR) effect. The size of nanoparticles plays a crucial role in this process, with studies indicating that particles smaller than 200 nm can passively accumulate in tumor tissue [65]. The NPs developed in this study fall within this size threshold, with measured sizes of 146.9 and 141.1 nm, thus meeting the criteria for effective EPR-based tumor targeting.

Encapsulating the extract in PLGA offers the advantage of controlled degradation. Lactic acid, being more hydrophobic than glycolic acid, absorbs less water, leading to slower degradation of PLGA. This slower degradation is accompanied by a similarly prolonged release of the active compound [23]. In our study, the cytotoxic effect of PLGA nanoparticles containing the methanolic extract of *V. greggii* (NPs F-Me) on HeLa cells showed a decrease in cell viability starting at 300 μg/mL, with an IC₅₀ of 271.37 μg/mL. This effect may be attributed to the composition of the nanoparticles (PLGA 75:25), where the higher proportion of lactic acid could minimize extract release within the first 24 h, the timeframe evaluated in this study. These findings align with those reported by Amjadi et al. (2013) [29], where PLGA 75:25 nanoparticles loaded with doxorubicin exhibited a 14.70% release of the total drug after 24 h and 62.22% release after 20 days. This suggests that extending the exposure time of cells to nanoparticles loaded with *V. greggii* could enhance cytotoxicity further.

In 2018, the anticancer potential of a sesquiterpene lactone, Calein C, isolated from *Calea pinnatifida* (*Asteraceae*), was evaluated on the MCF-7 cell line. Immunofluorescence analysis revealed that cells treated with Calein C displayed monopolar mitotic spindles, asymmetric bipolar spindles, and multinucleated cells. Its mechanism of action as a cell proliferation inhibitor was linked to the downregulation of genes encoding proteins (PLK1 and CDKN1A) essential for mitotic progression [66].

The antioxidant, antibacterial, antiviral, and cytotoxic potential of bioflavonoids, either nanoencapsulated or formulated in NPs, such as rutin—a compound identified in this study—have also been observed [67,68,69]. The bactericidal effect of rutin is attributed to its quorum sensing (QS) inhibitory activity against *Pseudomonas aeruginosa* and *Staphylococcus aureus* [68]. Cytotoxicity tests using PCL-PEG-based NPs loaded with rutin demonstrated dose- and time-dependent cytotoxic effects against MDA-MB-231 and Skov-3 cells. This enhanced cytotoxicity was associated with the upregulation of caspase-8, -9, -3, and Bax genes compared to cells treated with non-encapsulated rutin [69].

Additionally, a study in which the stilbenoid polydatin was nanoencapsulated in PLGA-NPs demonstrated that this formulation exhibited cytotoxic activity against KB cells, effectively inducing oxidative stress and initiating cellular damage leading to apoptosis [70]. This highlights the potential of plants from the *Asteraceae* family as chemotherapeutic agents, emphasizing the presence of sesquiterpene lactones and flavonoids in *V. greggii*, as well as the promising use of NPs.

Nuclear DNA is organized with structural proteins into dynamic higher-order chromatin structures, which regulate gene expression throughout the cell cycle and during cell differentiation. During mitotic chromatin condensation, most DNA-associated processes, such as transcription and replication, are halted [71]. Nuclear condensation is characterized by intense blue fluorescence, indicating DNA damage and serving as a marker for apoptosis [72]. Uniform nuclear staining, on the other hand, suggests an absence of genetic material damage. These features can be observed through DAPI staining [71].

Our findings corroborate these observations, as HeLa cells treated with F-Me and NPs F-Me at concentrations of 6 and 300 μg/mL exhibited intense blue fluorescence due to chromatin condensation (pyknosis), cell shrinkage, and a rounded morphology [73]. Additionally, reduced cell confluence created noticeable gaps between neighboring cells.

Furthermore, the free and nanoencapsulated methanolic fraction of *V. greggii* was evaluated for its hemolytic effect on human erythrocytes. F-Me showed no hemolytic activity at concentrations of 100–1000 μg/mL, while NPs F-Me were hemolytic only at 800 and 1000 μg/mL. A previous study evaluated the hemolytic effect of an infusion of *Vernonia amygdalina* leaves on human erythrocytes. The infusion induced hemolysis in SS genotype erythrocytes, moderate hemolysis in AS genotype erythrocytes, and no significant hemolysis in AA erythrocytes [66].

## 5. Conclusions

The methanolic fraction of *V. greggii* (F-Me), both in its free form and when nanoencapsulated, demonstrated significant antineoplastic activity against HeLa cells. Among the tested samples, this fraction exhibited the highest cytotoxicity compared to the crude extract and the *n*-hexane and aqueous fractions.

Future research should focus on elucidating the molecular mechanisms underlying the cytotoxic effects reported in this study.

## Figures and Tables

**Table 1:** Preliminary phytochemical analysis and extraction yields of *V. greggii* extract and fractions.

Test	Chemical Group	ME	F-Me	F-He	F-Ac
Liebermann-Burchard	Sterols, Triterpenes	++	++	+	−
NaOH	Coumarins	+	++	++	+++
Baljet	Sesquiterpene Lactones	++	+++	−	++
Sulfuric acid	Quinones	−	−	−	+++
Saponins	Saponins	−	−	−	−
Shinoda	Flavonoids	+++	−	−	+++
Ferric Chloride	Tannins	++	+++	−	+++
Anthrones	Carbohydrates	++	+++	+	+++
Dragendorff	Alkaloids	−	−	−	−
Extraction yields	%	32.96	0.34	17.98	16.65

Negative (−); Slightly positive (+); Positive (++); Highly positive (+++); %: Percent yield.

**Table 2:** UV–Vis and MS data for compounds identified in the F-Me extract.

Retention Time (min)	λmax (nm)	[M-H]^−^	Fragment Ions	Compound
1.9	324	353	191, 179, 135, 173	Caffeoylquinic acid derivative
2.6	325	353	135,173, 179, 191	Caffeoylquinic acid derivative
3.5		465	447, 375, 345, 327, 287, 201	Quercetin glycoside
4.0	324	353	191, 179, 135, 173	Caffeoylquinic acid derivative
5.1	225, 292	653	635, 507, 491, 449, 359, 329, 301	Dimethylquercetin-3-O-dihexoside
6.8	325	353	191, 179, 173, 135	Caffeoylquinic acid derivative
10.1	205, 256, 317	431	311, 341, 283	Vitexin
10.5	202, 269, 319	609	301, 300, 271, 255	Rutin
10.9	204, 256, 335	463	301, 300, 271, 255, 179, 151	Isoquercitrin
11.6	328	515	353, 179, 335, 203, 191, 255, 299	Dicaffeoylquinic acid derivative
11.7	328	515	353, 191, 335, 179, 434, 173	Dicaffeoylquinic acid derivative
12.4	328	515	353, 203, 299, 255, 179, 317, 173	Dicaffeoylquinic acid derivative
12.9	326	515	353, 203, 299, 179, 173, 335, 255	Dicaffeoylquinic acid derivative
16.8	329	353	191, 179, 263, 335, 272	Caffeoylquinic acid derivative
17.0	329	353	179, 191, 135, 161, 251	Caffeoylquinic acid derivative
17.3	328	353	191, 179, 161, 355, 135, 173	Caffeoylquinic acid derivative

**Table 3:** Cytotoxicity effect of *Vernonia greggii* fractions against HeLa and HaCat cells.

Treatment	IC_50_ (μg/mL)		SI
	HeLa	HaCat	
EM	268.08 ± 31.63 ^c^	ND	-
F-Me	4.33 ± 28.12 ^a^	4.78 ± 24.23 ^a^	1.10
F-Ac	237.02 ± 43.78 ^c^	352.46 ± 29.05 ^c^	1.48
F-He	103.04 ± 45.12 ^b^	158.89 ± 26.68 ^b^	1.54

Data are presented as mean ± standard deviation (SD) of IC₅₀ values (μg/mL) for each treatment, analyzed by one-way ANOVA with Tukey’s post hoc test for the comparison of means. Different letters within each column indicate significant differences between treatments, as determined by Tukey’s test. ND: Not determined, as it did not produce a significant effect.

**Table 4:** Characterization of nanoparticles.

	Size (nm)	PDI	ζ Potential (mV)	ee %	dl %
NPs F-Me	146.90 ± 1.159	0.103 ± 0.010	−23.30 ± 8.4	64.21	11.38
PLGA NPs	141.10 ± 0.815	0.096 ± 0.013	−20.20 ± 9.82	-	-

Characterization of methanolic fraction-loaded nanoparticles (NPs F-Me) and unloaded nanoparticles (PLGA NPs). Values for particle size (nm), polydispersity index (PDI), zeta potential (ζ, mV), encapsulation efficiency (ee, %), and drug loading percentage (dl, %) are presented as mean ± SD.

**Table 5:** Cytotoxicity of the NPs against HeLa and HaCat cells.

Treatment	IC_50_ (μg/mL)		SI
	HeLa	HaCat	
NPs F-Me	289.94 ± 39.39	274.05 ± 35.65	0.94

The data are presented as mean ± SD of IC_50_ in μg/mL.

**Table 6:** Hemolytic activity of F-Me and NPs F-Me.

Concentration	Hemolysis Percent (%)	
(μg/mL)	F-Me	NPs F-Me
Negative control	0.00	0.00
Positive control	100.00	100.00
100	0.00 ± 0.22	0.00 ± 0.06
200	0.17 ± 0.48	0.00 ± 0.21
400	0.00 ± 0.10	0.00 ± 0.33
600	0.49 ± 0.89	0.00 ± 0.42
800	0.00 ± 0.45	14.18 ± 0.46
1000	0.13 ± 1.22	32.23 ± 0.70

Data are mean ± SD of the percentage (%) of hemolytic activity.

## Data Availability

The original contributions presented in the study are included in the article, further inquiries can be directed to the corresponding authors.

## Abbreviations

%: Percent; °C: Celsius degrees; µg/mL: Micrograms per milliliter; μm: Micrometer; µM: Micromoles; AAPH: 2,2′-azobis (2-methylpropionamidine) dihydrochloride; ANOVA: Analysis of Variance; AP: Aqueous Phase; ATCC: American Type Culture Collection; AU: Absorbance Units; CHCl₃: Chloroform; CO_2_: Carbon Dioxide; CV: Cell Viability; DAPI: 4′,6-diamidino-2-phenylindole; dl%: Drug Load Percentage; DMEM: Dulbecco’s Modified Eagle Medium; ee%: Encapsulation Efficiency Percentage; EXE: Encapsulated Extract’s Mass; EXI: Initial Extract’s Mass; Ext: Extract; F-Ac: Aqueous Fraction; F-He: Hexanic Fraction; F-Me: Methanolic Fraction; FBS: Fetal Bovine Serum; GmbH: Gesellschaft mit beschränkter Haftung; HaCat: Human Epidermal Keratinocytes; HeLa: Human Cervical Cancer Cells; HPLC: High-Performance Liquid Chromatography; IC₅₀: Half Maximal Inhibitory Concentration; LPS: Lipopolysaccharide; OP: Organic Phase; PCS: Photon Correlation Spectroscopy; PDI: Polydispersity Index; PLGA: Poly(lactic-co-glycolide); ME: Methanolic Extract; MeOH: Methyl Alcohol; MN: Total Nanoparticle’s Mass; MTT: 3-(4,5-dimethylthiazol-2-yl)-2,5-diphenyltetrazolium bromide; nm: Nanometer; mV: Millivolts; NaOH: Sodium Hydroxide; NPs: Nanoparticles; OD: Optical Density; SD: Standard Deviation; SI: Selectivity Index; UV: Ultraviolet; UV–Vis: Ultraviolet–Visible; ζ: Zeta Potential.
